# β-Cyclodextrin-Based Supramolecular Imprinted Fiber Array for Highly Selective Detection of Parabens

**DOI:** 10.3390/ijms231810753

**Published:** 2022-09-15

**Authors:** Zhimin Liu, Qingqing Zhou, Dan Wang, Yunli Duan, Xuehua Zhang, Yi Yang, Zhigang Xu

**Affiliations:** Department of Chemistry, Faculty of Science, Kunming University of Science and Technology, Kunming 650500, China

**Keywords:** β-cyclodextrin, supramolecule, molecular imprinting, fiber array extraction, parabens

## Abstract

A novel high-throughput array analytical platform based on derived β-cyclodextrin supramolecular imprinted polymer (SMIP) fibers was constructed to achieve selective enrichment and removal of parabens. SMIP fiber arrays have abundant imprinting sites and introduce the host–guest inclusion effect of the derived β-cyclodextrin, which is beneficial to significantly improve the adsorption ability of fiber for parabens. Upon combination with HPLC, a specific and sensitive recognition method was developed with a low limit of detection (0.003–0.02 µg/L, S/N = 3) for parabens analysis in environmental water. This method has a good linearity (R > 0.9994) in the linear range of 0.01–200 µg/L. The proposed SMIP fiber array with high-throughput adsorption capacity has great potential in monitoring water pollution, which also provides a reliable reference for the analysis of more categories of pharmaceutical and personal care product pollutants.

## 1. Introduction

Parabens are a class of antibacterial preservatives commonly used in pharmaceuticals and personal care products (PPCPs) to extend the shelf life of products. However, parabens have been shown to have estrogenic activity and have a major impact on human health by interfering with the metabolism of the endocrine system [1,2]. Humans may be exposed to parabens through various routes including ingestion, inhalation, and dermal contact [3]. Recently, many studies have pointed out that low concentrations of parabens still increase the risk of certain diseases, such as decreased fertility [4], obesity [5], and gestational diabetes [6]. Water, an essential part of our daily life, has also become “reservoir” of parabens, which have caused irreversible damage to the aquatic ecosystem. Thus, monitoring and evaluating the qualities of parabens are necessary.

Molecularly imprinted polymers (MIPs), which were initially proposed by Wulf in 1972, can mimic the recognition mechanism of natural antibodies and antigens [7]. MIPs are synthetic receptors for a targeted molecule. The template molecule is removed from the polymer host, leaving a target-specific cavity available for rebinding [8]. Specific binding cavities of MIPs complementary to the structure, size, and shape of the template molecule can selectively rebind to analytes in complex matrices [9,10]. Synthesized MIPs can be formed into various morphological structures, such as bulk materials, nanomaterials, nanocomposites, and thin films, which will further broaden the range of applications [11]. At present, MIPs have been applied in chemical sensing [12], drug delivery [13,14], biological analysis [15,16,17], separation science [18,19], food detection [20,21], and environmental pollution monitoring [22,23]. Meanwhile, the combination of molecular imprinting technology and microextraction technology has achieved rapid development [24]. Molecularly imprinted solid phase microextraction (MIP–SPME) technology has been widely used in sample pretreatment [25]; it has been applied to the analysis and determination of parabens in environmental water [26,27,28,29,30]. Unfortunately, the hydrogen bond of molecular recognition is easily affected by the water medium. Thus, the recognition ability of molecularly imprinted polymers for the detection of pollutants in the water environment is reduced. Moreover, developing a highly sensitive molecularly imprinted solid phase microextraction polymer is essential for the analysis of parabens in water samples. In our previous study [30], we firstly presented supramolecularly imprinted polymeric solid phase microextraction coatings to recognition for nitrophenols and bisphenol A in real water samples. These polymers possess inclusion interactions from β-cyclodextrin cavities and hydrogen-bonding interactions from molecular imprinting which performed highly selective recognition in complex real water sample with sensitive detection limits. Furthermore, supramolecularly imprinted polymeric is applied to determination of parabens in environmental water need our further study.

The combination of supramolecular host and guest compounds with molecular imprinting technology has been proven to be a good specific adsorbent in aqueous [30,31,32,33]. Cyclodextrin (CD) is the most common supramolecular host compound; it is a hollow truncated cone with a hydrophobic cavity and hydrophilic rims. It can form host–guest clathrates with various molecules, which provide more favorable interactions for the adsorption of target analytes by polymer materials in aqueous media. Thus, β-CD-based supramolecular imprinting (SMIP) fibers are based on various molecular interactions, such as hydrophobic, electrostatic, hydrogen bonding, and host–guest inclusion. Furthermore, molecularly imprinted polymer array device strategy has been proven to significantly improve the high-throughput enrichment performance of various analytes [34,35,36].

In this study, five types of β-CD derivatives were used as supramolecular functional monomers and propylparaben (PP) as the template molecule to prepare SMIP fibers. In addition, three common functional monomers of acrylamide (AM), methacrylic acid (MAA), and 4-vinylpyridine (4-VP) were used to prepare MIPs as comparator. Under the same conditions, corresponding non-molecularly imprinted polymer (NIP) fibers were synthetized in the absence of the template. The preferred fibers are used to further construct SMIP fiber arrays with high adsorption flux by evaluating sorption properties of the fibers to select best SMIP. Finally, combined with high-performance liquid chromatography, a method for the high-sensitivity analysis and detection of parabens environmental estrogens in environmental water samples is developed.

## 2. Results and Discussion

### 2.1. Characterization of Polymer Materials

#### 2.1.1. FTIR

The functional group and composition of imprinted polymer fiber coatings were studied using FTIR. After removing the template molecules, the SMIP, AM-NIP, and their corresponding NIP fiber coatings were pulverized and ground for FTIR measurement. As shown in Figure S1, the results indicate that SMIP and AM-MIP have similar chemical groups with their corresponding NIP. The FTIR absorption peaks of the same functional monomer MIP and NIP fiber coatings are very similar. This finding could indicate that the template molecule is indirectly involved in the polymerization reaction and only interacts with the functional monomer through weak hydrogen bonds.

#### 2.1.2. SEM Image 

**Figure 1:**
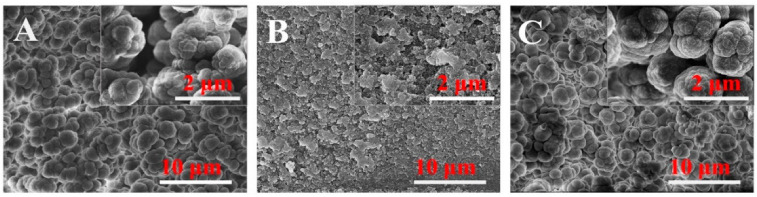
SEM images of three fiber coatings. (**A**) SMIP; (**B**) AM-MIP; (**C**) SNIP.

The surface morphology of SMIP, AM-MIP, and SNIP fiber coatings were studied by SEM. Figure 1 show the morphological characteristics of the three polymer fiber coatings at different magnifications. The results show that the surface of the polymer coating with cyclodextrin as a functional monomer is rough and wrinkled, grouped together in small clusters, and leaving certain phenotypic gaps. The presence of the unique cavity of the cyclodextrin creates a host–guest inclusion with the target molecule and creates a synergistic extraction with the specific recognition of the imprinted polymer, which improves the adsorption capacity of the target analytes. Furthermore, SMIP coating clusters form particles larger than SNIP particles, thereby increasing the recognition site and facilitating the adsorption of target molecules.

#### 2.1.3. N_2_ Adsorption–Desorption Characterization

**Figure 2:**
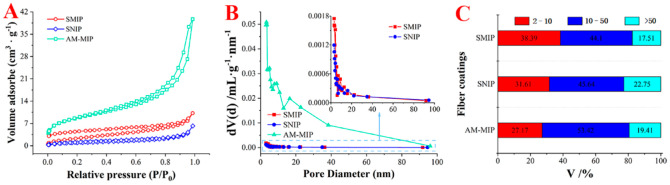
BET Characterization for three fiber coatings. (**A**) N_2_ adsorption–desorption isotherms curve; (**B**) Pore size distribution profiles; (**C**) Different pore size ratio analysis.

Figure 2 shows the N_2_ adsorption–desorption isotherms and pore size distribution profiles for SMIP, SNIP, and AM-MIP. The adsorption curves of SMIP, SNIP, and AM-MIP exhibited type-Ⅳ isotherm. CD polymer and AM polymer coatings have significant differences in pore size distribution curves and surface area. Pore size was measured with the Brunauer–Emmett–Teller (BET) method, and the ratio of different pore size distributions is shown in Figure 2C. The ratio of pore size distributions differed. In particular, the ratio of pore size ranged from 2.0 nm to 10.0 nm in SMIP, SNIP, and AM-MIP, that is, 38.39%, 31.61%, and 27.17%, respectively. The surface areas were 13.081, 5.743, and 437.196 m^2^/g, respectively. The pore volume and BET surface area of the SMIP coating were different from those of SNIP and AMP-MIP (Table S1). These differences result in different adsorption properties for each fiber coating.

### 2.2. Theoretical Calculation Verification

Various molecular modeling and theoretical calculations in molecular imprinting technology use the DFT method to calculate the binding energy (ΔE) between the template molecule and the functional monomer. Generally, the lower energy value indicates stronger intermolecular interaction. This finding shows that the composite system of the template molecule and the functional monomer is more stable, and the prepared MIP has excellent performance [37,38,39,40].

**Figure 3:**
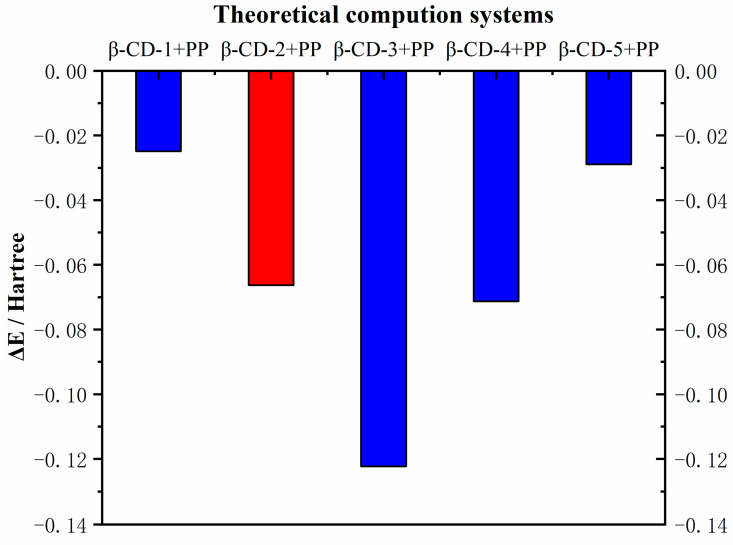
Calculated values of template–monomer interaction energies by theoretical calculation.

Results showed that β-CD-3+PP had the highest binding energy than that of the other complexes (Figure 3). However, cracking of the material synthesized by β-CD-3 functional monomer seriously affected the evaluation of the extraction performance. β-CD-2+PP and β-CD-4+PP had similar binding energies, and the extraction experiments showed that β-CD-2 had the best extraction performance when used as the functional monomer. On the contrary, the lowest bonding energy value of −0.0663 was achieved by β-CD-2+PP complexes (Table 1). The results also verify the findings of the extraction experiments to select the best functional monomer. Therefore, β-CD-2 was selected as the best functional monomer for the subsequent experiments. 

### 2.3. Optimization of Extraction Conditions for SMIP Fiber Array

#### 2.3.1. Desorption Solution Optimization

Methanol, methanol: acetic acid (9:1, *v*/*v*), acetonitrile and acetonitrile: acetic acid (9:1, *v*/*v*), and acetonitrile: water (1:1, *v*/*v*) (mobile phase) were selected as desorption solvent for optimization. The SMIP fiber array was immersed in 50 mL of PP aqueous solution at a concentration of 50 μg/L with extracted stirring for 120 min. Then, it was desorbed in 0.25 mL methanol by sonication for 5 min. The results show that the five desorption solvents have a certain desorption capacity for PP (Figure S2). The desorption efficiency of PP was the lowest when the mobile phase was desorption solvent, and the desorption capacity was the highest when methanol was used as a desorption solvent; thus, methanol was the best desorption solvent.

#### 2.3.2. Solution pH Optimization

The pH of aqueous solution can affect the existence form of molecules, which in turn affect the extraction efficiency. Therefore, the influence of the pH value of the extraction solution on the extraction amount of PP adsorbed by SMIP fiber array was investigated. The pH of the extraction solution was adjusted by adding hydrochloric acid or sodium hydroxide for optimization experiments. The pKa of parabens is 8.23 ± 0.15, and PP may undergo hydrolysis when the pH is higher than 9. Thus, we study the pH range of the extraction solution at 4.0–9.0. The results are shown in Figure S3, the amount of PP extracted increased slightly with the increase in pH. When the pH of the extraction solution was 8.0, the adsorption of the target analyte by the SMIP fiber array was most favored and reached equilibrium.

#### 2.3.3. Ionic Strength Optimization

In extracted solutions containing different mass fractions of NaCl, the solubility of the analytes differs due to the salting effect and the dissolved salt effect, which causes SMIP fiber array to extract different amounts of PP. Therefore, NaCl extraction solutions with mass fractions of 0%, 5%, 10%, 15%, and 20% were prepared to investigate the effect of ionic strength on the extraction of PP by SMIP fiber array. As shown in Figure S4, the extraction amounts of PP slightly increased with the increase in the NaCl mass fraction, and the maximum extraction amount of PP was obtained when the NaCl mass fraction was 15%. Subsequently, the high content of NaCl may increase the viscosity of the solution, which is not conducive to the adsorption of the target substance by SMIP fiber array. Therefore, the best ionic strength was 15% NaCl in the extraction solution.

#### 2.3.4. Extraction Time Optimization

In the dynamic adsorption process of the SMIP fiber array to PP, adsorption time plays a critical role in affecting the extraction amount of the target compound. Here, 30–300 min (interval 30 min) was set as the extraction time for optimization experiments to investigate the effect of extraction time on the extraction amount of PP adsorbed by SMIP fiber array. The results are shown in Figure S5. The SMIP fiber array exhibited significant increase in the amount of PP extracted until it reached equilibrium at 210 min. Therefore, 210 min was the best extraction time.

#### 2.3.5. Desorption Time Optimization

Desorption time also affects the desorption efficiency of the SMIP fiber array. If the desorption time is extremely short, then the analyte becomes incompletely desorbed. At the same time, if the desorption time is extremely long, then desorption efficiency has already reached its peak, causing certain damage to the material. To obtain a desorption time suitable for the SMIP fiber array, the time is set to 3, 5, 7, 10, and 15 min to carry out the desorption time optimization experiment. Figure S6 shows that the extraction amount of PP reached an equilibrium value at 5 min, the desorption time increased, and the extraction amount of PP was almost unchanged. Therefore, 5 min was selected as the most suitable desorption time.

### 2.4. Selectivity Adsorption Experiment of SMIP Fiber Array

**Figure 4:**
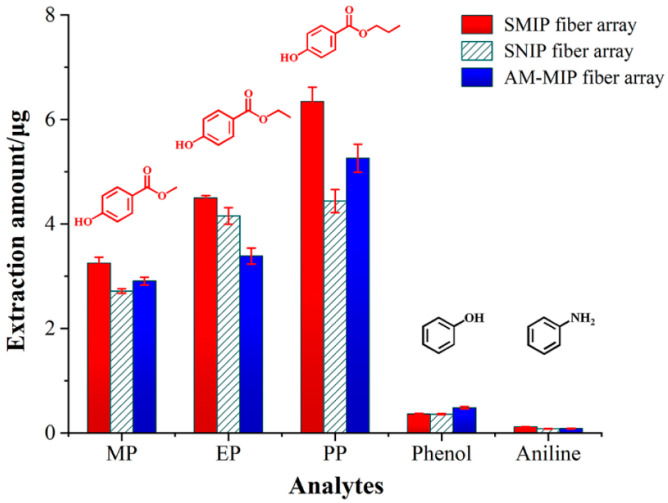
Selectivity study of three fiber arrays for different analytes.

Two structurally related compounds (MP and EP) and two other coexistences (phenol and aniline) are selected as interferences to investigate the selectivity of SMIP fiber array toward PP. The adsorption experiment was carried out under optimized extraction conditions, and the extraction concentration of each analyte was 200 μg/L. As shown in Figure 4, the extraction amount of SMIP fiber array for PP (6.35 µg) is much higher than those of other competitive compounds. The SMIP fiber array exhibits a certain adsorption capacity toward MP (3.25 µg) and EP (4.50 µg) due to its highly similar structure to PP. In addition, the extraction amounts of SMIP fiber array for phenol and aniline are much lower than those of MP and EP due to the structural dissimilarity to PP. These results imply that the imprinted sites remain crucial for specific recognition of SMIP fiber array.

To further study the selectivity of SMIP fiber array for PP, Table 2 displays the calculated K_d_, IF, and EF values. The corresponding IF value of 1.5 for PP and the EF value of 207.21 suggest that the SMIP fiber array possesses high selectivity and enrichment ability for PP. Furthermore, the parameters of extraction rate and desorption rate were evaluated, as shown in Table S2. 

### 2.5. Static Adsorption Experiment

**Figure 5:**
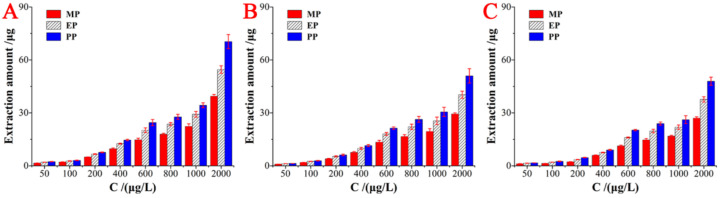
Extraction capacity for three analytes (**A**) SMIP fiber array; (**B**) SNIP fiber array; (**C**) AM-MIP fiber array.

To further investigate the adsorption characteristics of SMIP fiber array to target analytes. The static adsorption performance was studied under the optimal extraction conditions. Figure 5 shows the static adsorption of SMIP, SNIP, and AM-MIP fiber array toward three parabens. The extraction amounts gradually increased as the concentration of all analytes increased. Meanwhile, SMIP fiber array showed greater extraction amount than the two other fiber arrays under the same concentration. To reveal the superiority of the adsorption capacity of the SMIP fiber array, the adsorption data are further applied to the Scatchard curve to obtain the binding site and the theoretical maximum binding capacity; they are calculated by the Scatchard Equation (1), as follows:

(1) $$\frac{Q}{C_{e}}=\frac{{(Q}_{\max}\;-\;Q)}{K_{D}}$$

where Q and Qmax are the experimental adsorption capacity and theoretical maximum adsorption capacity (mmol/g) of the fiber array for the analyte, respectively. C_e_ (mmol/L) is the concentration of the analyte at equilibrium, and K_D_ is the dissociation constant (mmol/L) of the Scatchard curve. K_D_ and Qmax are important parameters.

**Figure 6:**
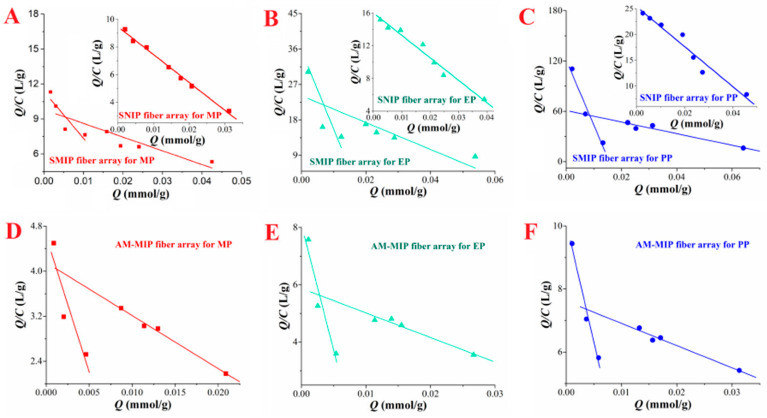
Scatchard fitting curve. (**A**–**C**). SMIP fiber array and SNIP fiber array for the adsorption of three parabens; (**D**–**F**). AM-MIP fiber array for the adsorption of three parabens.

The Scatchard curve is shown in Figure 6. SMIP and AM-MIP selective adsorption PP, the linear regression equations are Q/C_e_ = −1008.2002Q + 71.66029 (R^2^ = 0.9895), Q/C_e_ = −86.1280Q + 7.8561 (R^2^ = 0.9605), respectively. SNIP absorb PP the linear regression equation is Q/C_e_ = −387.8499Q + 25.39308 (R^2^ = 0.9448). The SMIP fiber array has high-affinity sites and low-affinity sites in the adsorption of three parabens. This finding demonstrates that SMIP has dual recognition effect on the target analytes compared with the SNIP fiber array, thereby obtaining superior adsorption performance. The Scatchard fitting parameters are further shown in Table 3. The K_D_ and Q_max_ values corresponding to the high affinity sites of the SMIP fiber array for PP are 1.29 × 10^−4^ mmol/L and 1.56 × 10^−2^ mmol/g, respectively. The K_D_ and Q_max_ values corresponding to the low-affinity sites were 9.92 × 10^−4^ mmol/L and 7.11 × 10^−2^ mmol/g, respectively. The SNIP fiber array has only low affinity sites for PP adsorption, and the corresponding K_D_ and Q_max_ values are 2.58 × 10^−3^ mmol/L and 6.55 × 10^−2^ mmol/g, respectively. In addition, the Q_max_ of the AM-MIP fiber array to the template molecule PP is 1.33 × 10^−3^ mmol/g, which is much smaller than the maximum adsorption capacity of the SMIP fiber array to PP.

### 2.6. Adsorption Performance Comparison with Commercial PA Fiber Array

**Figure 7:**
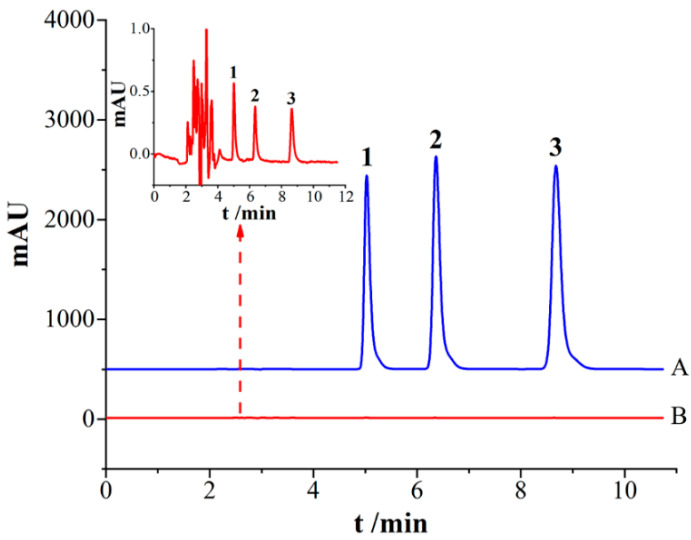
Chromatograms of PA commercial fiber array and SMIP fiber array for extraction PBs. A—SMIP fiber array; B—PA fiber array; 1. MP; 2. EP; 3. PP.

A self-assembled PA commercial fiber array was compared with the supramolecular imprinted fiber array to further study the adsorption performance of the SMIP fiber array. The results show that the commercial coating can only extract the target analyte at a high concentration. Furthermore, the adsorption amounts of parabens extracted by the SMIP fiber array were 3777–6174 times greater than the extracted PA commercial fiber array (Figure 7). This finding indicates that the self-made supramolecular fiber array has significantly superior adsorption capacity for parabens.

### 2.7. Longevity Evaluation

**Figure 8:**
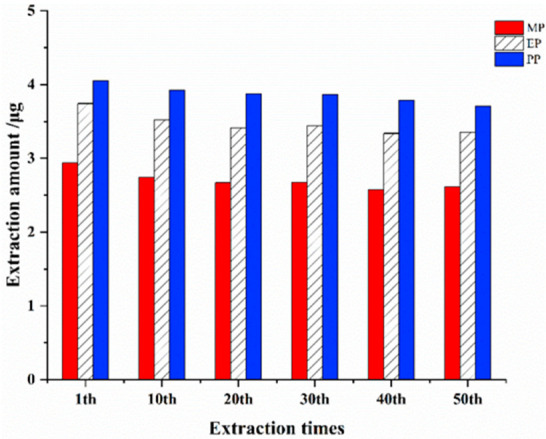
Evaluation of the service life of SMIP fiber array.

To study the recycling times of SMIP fiber array, mixed solution with concentration of 100 μg/L of MP, EP, and PP was extracted under the optimal extraction conditions, and adsorption and desorption were repeated for 50 cycles. The result in Figure 8 shows that after 50 times of recycling, the extraction amount of the three target analytes could still be maintained at 87.97–91.51%, whereas the RSD is not higher than 4.65%. The results indicated that SMIP fiber would insignificantly reduce the extraction amount of target analyte after 50 times of use. Therefore, the results indicate that the SMIP fiber array has a satisfactory longevity and can be used for long-term separation and enrichment of target analytes.

### 2.8. Application in Environmental Water Samples

SMIP fiber array was applied to the determination of three parabens in two environmental water samples. The matrix matching method was used to obtain the calibration curve. The three parabens have good linearity (R > 0.9994) in the linear range of 0.01–200 µg/L, and the limit of detection (LODs, S/N = 3) was 0.003–0.02 µg/L (Table 4). The established method was applied to the detection of two environmental water samples, shown on Figure S7.

The results showed that the target analytes were undetected in the two environmental water samples without material processed. After SMIP fiber array processing, MP and PP were detected at sampling site 2 at concentrations of 0.11 and 0.06 μg/L, respectively. In addition, the accuracy and precision of the method were further verified by the standard addition and recovery experiment. Three concentrations of low, medium, and high were added to the actual water sample, that is, the mixed solutions of MP, EP, and PP with concentrations of 1, 5 and 100 μg/L. As shown in Table 5, the recovery of standard addition was between 80.23% and 117.36%, and the RSD was less than 8.17%.

### 2.9. Method Comparison

Moreover, we compared this work with some previously reported tests for paraben endocrine disruptors. As shown in Table 6, the SMIP fiber array based on cyclodextrin supramolecular host compound as functional monomer has many specific recognition cavities and host–guest inclusion cavities in the imprinting polymer. This condition is conducive to the selective high-throughput adsorption and separation of paraben esters, thereby producing excellent detection sensitivity and not requiring expensive equipment. The detection limit of SMIP fiber array for MP, EP, and PP is more than three times higher than other reported methods, including MIP method.

## 3. Materials and Methods

### 3.1. Materials

Methylparaben (MP, >99%), ethylparaben (EP, >99%), and propylparaben (PP, >99%) were purchased from Aladdin. Analytical grade phenol and aniline were obtained from Guangdong Guanghua Chemical Factory Co., Ltd. Mono-(6-mercapto-6-deoxy)-β-cyclodextrin was obtained from Shandong Binzhou Zhiyuan Biological Technology Co., Ltd. (Bingzhou City, China). The other derived β-CDs have been synthesized in our previous report [37]. Analytical grade acrylamide (AM) and azobisisobutyronitrile (AIBN) were purchased from Tianjin Kaixin Chemical Industry Co., Ltd. (Tianjin City, China). Ethylene glycol dimethacrylate (EDMA) was purchased from Guangzhou Dike Composite Material Technology Co., Ltd. (Guangzhou City, China). Chlorinated sodium (NaCl), analytical grade methanol, and acetonitrile were obtained from Tianjin Zhiyuan Chemical Reagent Co., Ltd. (Tianjin City, China). HPLC grade methanol and acetonitrile were purchased from BCR International Trading Company (New Jersey, USA). Industrial nitrogen was obtained from Kunming Rongtai Industry and Trade Co., Ltd. (Kunming City, China). Hydrochloric acid (HCl), sodium hydroxide (NaOH), and dimethyl sulfoxide (DMSO) were purchased from Tianjin Fengchuan Chemical Reagent Technology Co., Ltd. (Tianjin City, China). Pure water was purchased from Hangzhou Wahaha Group Co., Ltd. (Hangzhou City, China). Two types of glass capillary (1.8–2.2 and 0.9–1.1 mm diameter, 100 mm length) were obtained from the Medical Science Instrument Plant of West China University.

### 3.2. Apparatus

HPLC (Dionex Ultimate 3000, Germering City, Germany.) with C18 chromatographic column (5 μm, 250 mm × 4.6 mm i.d., J&K Scientific Ltd., Beijing, China) was used to detect analytes. The characteristic functional groups were characterized by Fourier-transform Infrared (FTIR) spectrometry (TENSOR-27, Brucker, Germany). The surface appearance of polymer coatings was characterized by scanning electron microscopy (SEM, Nova Nano SEM 450FEI-IMC, Oregon, USA). Then, the surface areas and porous properties of polymer coatings were measured by the Brunauer–Emmett–Teller (BET) method.

### 3.3. Preparation of Cyclodextrin Supramolecularly Imprinted Polymer Fibers

#### 3.3.1. Functional Monomer of Cyclodextrin Derivatives

**Figure 9:**
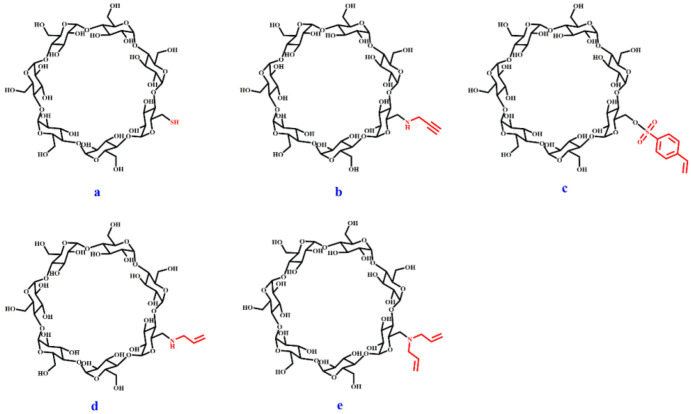
Molecular structure of five functional monomers of cyclodextrin derivatives. (**a**) mono-(6-mercapto-6-deoxy)-β-CD (β-CD-1); (**b**) mono-(6-*N*-propargyl-6-deoxy)-β-CD (β-CD-2); (**c**) mono-(6-*O*-p-vinylbenzene sulfonyl-6-deoxy)-β-CD (β-CD-3); (**d**) mono-(6-*N*-allylamino-6-deoxy)-β-CD (β-CD-4); (**e**) mono-(6-*N*-diallylamine-6-deoxy)-β-CD (β-CD-5).

The structure of five derived β-CDs is shown in Figure 9. Compound (a), named as mono-(6-mercapto-6-deoxy)-β-CD (β-CD-1), could be obtained by purchasing commercially available product. Compounds of (b), (c), (d), and (e) were synthesized and characterized in our previous report [46]. They were used for β-cyclodextrin supramolecularly imprinted polymer fiber coatings. 

#### 3.3.2. SMIP Fiber Preparation

The MIP-SPME fibers were obtained using PP as the template molecule and five cyclodextrin derivatives as functional monomers. In addition, three commonly used functional monomers were selected to prepare MIP fibers for comparison. Fibers, prepared by in-situ polymerization under the conditions of 1 mmol template molecule and 4 mmol functional monomer, were fully dissolved in 20 mL DMSO, and the mixed solution was placed at room temperature for pre-polymerization for 8 h. Then, 60 mmol EDMA and 20 mg AIBN were added. After ultrasonic mixing, oxygen was removed by blowing nitrogen. Then, the solution was injected into a 1.8–2.1 mm glass capillary jacket and flowed by inserting a 0.9–1.1 mm glass capillary modified with a silanization reagent as the polymer coating substrate. The gap between the two capillary tubes was sealed with a rubber tape to isolate the oxygen. After polymerizing in a constant temperature water bath at 60 °C for 24 h, the external glass capillary was removed, and the polymer coating attached to the glass capillary surface was cut off to a length of 1.8 cm. Finally, the obtained MIP fiber was stirred and eluted in a methanol solution to remove template molecule.

#### 3.3.3. Optimization of Functional Monomer

Eight types of molecularly imprinted fiber coatings were prepared. An extraction experiment was conducted using MIP fiber for PP analysis to find the best functional monomer. The 50 mL PP solution with a concentration of 100 μg/L was extracted by MIP fiber for 120 min. Then, the fiber was ultrasonically desorbed with 0.25 mL methanol for 5 min. The desorption solution was then injected for HPLC detection. The results are shown in Table S3. Best adsorption performance could be obtained using mono-(6-*N*-propargyl-6-deoxy)-β-CD as the functional monomer. The supramolecularly imprinted polymer coating is also relatively stable in appearance without coating detachment. Subsequently, the SMIP was also constructed with this type of fiber to obtain great adsorption flux and high detection sensitivity. Three randomly selected monomers, methacrylic acid, acrylamide and 4-vinylpyridine, which are non-covalently bound to the template molecule. Acrylamide functional monomer, its corresponding imprinted polymer (AM-MIP) and the corresponding SNIP were used to construct the array as control.

### 3.4. Chromatography Analysis

The chromatographic column was C18 with the column temperature at 30 °C, and acetonitrile–water solution (50:50, *v*/*v*) was used as the mobile phase with a flow rate of 1 mL/min; the detection wavelength of parabens was 254 nm, and the injection volume of detection solution was 20 μL.

### 3.5. Construction of SMIP Fiber Array

**Figure 10:**
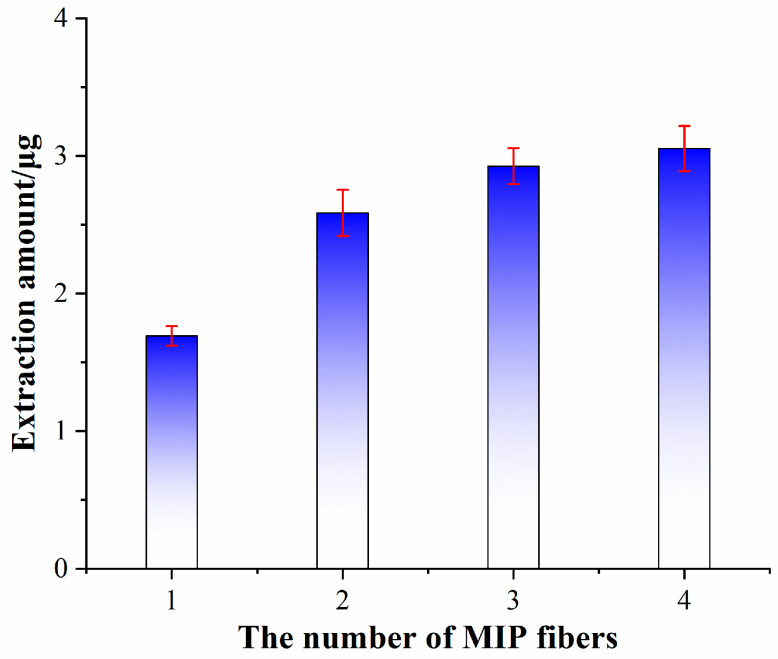
Effect of the number of MIP fibers on the extraction amounts.

Cyclodextrin-based supramolecular imprinting materials have been proven to have superior specific adsorption performance in aqueous media, but in actual water sample detection, the simultaneous high-throughput enrichment method for multiple target analytes still needs further development. We constructed SMIP fiber array by using mono-(6-*N*-propargyl-6-deoxy)-β-CD as functional monomer to further improve the adsorption flux. Two, three, and four SMIP fibers were used to construct an array analysis platform, and a single fiber was used as control (Figure 10). The extraction experiments revealed that the fiber arrays assembled by three supramolecular imprints reached an equilibrium value for PP extraction that was 1.7 times greater than the extraction of a single fiber. In addition, the amount of extraction did not change significantly as the number of fibers increased. Therefore, the SMIP fiber array assembled by three polymer fibers can be considered the best enrichment device. Meanwhile, SNIP and AM-MIP fiber arrays with three fibers were constructed as control.

### 3.6. Selectivity Adsorption Experiment

The structural analogues of the template molecule propyl paraben (PP), methyl paraben (MP), ethyl paraben (EP), as well as two nonstructural analogs phenol and aniline were selected for selective research. Under the optimal extraction conditions, the concentration of each analyte was determined at 100 μg/L. The chromatographic conditions of phenol and aniline were acetonitrile–water (50:50, *v*/*v*) as the mobile phase, the flow rate was 1 mL/min, the detection wavelength was 254 nm, and the column temperature was 30 °C. The extraction and desorption rates were calculated by formulas (1) and (2). The partition coefficient (K_d_, mL/g), imprinting factor (IF), and enrichment factor (EF) are used for evaluation to further study the selectivity and enrichment ability of fiber, and their values are calculated by formulas (4)–(6), as follows:

(2) $$\mathrm{Desorption}\;\mathrm{yield}\left(\%\right)=\frac{\;m_{1}}{m_{2}}\times 100\%$$

(3) $$\mathrm{Extraction}\;\mathrm{recovery}\left(\%\right)=\frac{m_{2}}{m_{0}}\times 100\%$$

(4) $$Q_{e}=\frac{V\left(C_{0}{-C}_{e}\right)}{m}$$

(5) $$K_{d}=\frac{Q_{e}}{C_{e}}$$

(6) $$\mathrm{IF}=\frac{Q_{e}\left(\mathrm{MIP}\right)}{Q_{e}\left(\mathrm{NIP}\right)}$$

(7) $$\mathrm{EF}=\frac{C_{f}}{C_{i}}$$

where m_1_ is the first desorption mass of the analyte in the coating; m_2_ is the total mass after multiple desorptions; m_0_ is the total mass prior to extraction. C_0_ (μg/L) is the initial concentration of each analyte; C_e_ (μg/L) is its equilibrium concentration; V (mL) is the volume of the extraction solution; m (mg) is the quality of the coating of the extraction material; Q_e_ (mg/g) is the equilibrium binding capacity of each analyte to the coating of the extraction material, Q_e_ (MIP) and Q_e_ (NIP) are the MIP and NIP adsorption capacities, respectively. C_i_ (μg/L) is the initial concentration prior to extraction, and C_f_ (μg/L) is the concentration of the analyte in the desorption solvent.

### 3.7. Theoretical Calculation

Density Functional Theory (DFT) method in quantum mechanics has low computational complexity; it is used in MIP design and mechanism interpretation simulation calculations. This method can calculate molecular bond energy [47], predict compound structure [48], and predict mechanism of action [49]. Therefore, we used DFT method to study the interaction strength between PP and five cyclodextrin functional monomers and three common functional monomers to verify the results of extraction experiments at the molecular level.

### 3.8. Application in Environmental Water Samples

Two environmental water samples were collected at the entrance and exit of Laoyu River and Wetland Park, respectively. The two water samples were filtered three times with suction, and then filtered with a 0.22 µm microporous filter. After removing the solid impurities, the water sample is stored in a refrigerator at 4 °C for subsequent use.

## 4. Conclusions

In summary, an SMIP fiber array was constructed using β-cyclodextrin-based supramolecularly imprinted polymers for the high-throughput enrichment and selective removal of three parabens endocrine disruptors in environmental water. SMIP fiber array not only has abundant imprinting sites but are also more conducive to derived β-cyclodextrin functional monomer interaction with parabens through the inclusion and hydrophobic interaction. In addition, the SMIP fiber array has a very superior enrichment capacity compared with the commercial PA fiber array; it has an enrichment factor of 3777–6174 times. SMIP fiber array also has good reusability for adsorption parabens at least 50 times. SMIP fiber array combined with HPLC was applied to the specific separation and detection of three parabens in river water samples. The established method has good accuracy and high sensitivity. This work provides a new strategy for selective, high-throughput, and high-sensitivity imprinted polymers as adsorbent materials, which have great potential in the selective enrichment and separation of PPCP pollutants in complex environmental water samples.

## Figures and Tables

**Table 1:** Δ Total DFT-D energy and Δ bonding E values for the templates and monomer complexes.

Complex	Total DFT-D Energy (T)/Hartree	Bonding (E)/Hartree	ΔE Bonding (E)/Hartree
PP	−613.4714	−4.5478	/
β-CD-1	−4996.1179	−27.8396	/
β-CD-2	−4367.7866	−26.0865	/
β-CD-3	−5128.8238	−28.2004	/
β-CD-4	−4485.5895	−27.5137	/
β-CD-5	−4367.7864	−26.0862	/
β-CD-1+PP	−5609.6230	−32.4123	−0.0249
β-CD-2+PP	−4981.3244	−30.7006	−0.0663
β-CD-3+PP	−5742.426	−32.8705	−0.1223
β-CD-4+PP	−5099.132	−32.1328	−0.0713
β-CD-5+PP	−4981.287	−30.6629	−0.0289

**Table 2:** Distribution coefficients (K_d_), imprinting factor (IF), and enrichment factor (EF) calculated from the selectivity study.

Analytes	K_dSMIP_	K_dSNIP_	K_dAM-MIP_	IF	EF_SMIP_	EF_SNIP_	EF_AM-MIP_
MP	5331.20	4383.21	3421.80	1.22	106.06	88.00	90.53
EP	7377.62	6700.53	3984.53	1.10	139.92	128.24	117.82
PP	9829.66	6774.45	6187.14	1.45	207.21	133.38	148.32
Phenol	594.28	579.94	566.84	1.02	12.94	12.55	16.59
Aniline	197.44	131.84	100.34	1.50	4.21	2.88	3.40

**Table 3:** Scatchard fitting parameter analysis.

Fiber Array	Analytes	Low-Affinity Sites		High-Affinity Sites	
		K_D_ (mmol/L)	Q_max_ (mmol/g)	K_D_ (mmol/L)	Q_max_ (mmol/g)
SMIP	MP	2.48 × 10^−3^	2.82 × 10^−2^	8.50 × 10^−3^	8.33 × 10^−2^
	EP	3.47 × 10^−3^	7.75 × 10^−2^	6.49 × 10^−4^	2.00 × 10^−2^
	PP	9.92 × 10^−4^	7.11 × 10^−2^	1.29 × 10^−^^4^	1.56 × 10^−2^
SNIP	MP	4.94 × 10^−3^	4.68 × 10^−2^	/	/
	EP	3.64 × 10^−3^	5.84 × 10^−2^	/	/
	PP	2.58 × 10^−3^	6.55 × 10^−^^2^	/	/
AM-MIP	MP	1.40 × 10^−2^	5.23 × 10^−2^	2.08 × 10^−3^	9.59 × 10^−3^
	EP	1.06 × 10^−2^	6.24 × 10^−2^	1.14 × 10^−3^	9.23 × 10^−3^
	PP	1.16 × 10^−2^	9.12 × 10^−2^	1.32 × 10^−3^	3.4 × 10^−3^

**Table 4:** Linear equation, lower limit of detection, and lower limit of quantification of the method.

Analytes	Linear Range (μg/L)	Linear Equation	R	LOD (μg/L)	LOQ (μg/L)
MP	0.01–200	y = 0.28216x + 0.14119	0.9994	0.003	0.01
EP	0.05–200	y = 0.4125x + 0.19874	0.9994	0.02	0.05
PP	0.05–200	y = 0.38869x + 0.23567	0.9994	0.02	0.05

**Table 5:** Detection of parabens in environmental water samples and analysis of recovery (n = 3).

Samples	Analytes	Found (μg/L)	Added 0.1 μg/L		Added 5 μg/L		Added 100 μg/L	
			Recoveries (%)	RSD (%)	Recoveries (%)	RSD (%)	Recoveries (%)	RSD (%)
Site 1	MP	ND	91.32	2.31	84.86	5.75	117.36	4.97
	EP	ND	97.63	4.60	85.54	3.71	106.73	1.82
	PP	ND	85.78	3.84	92.51	7.12	99.52	0.58
site 2	MP	0.11	81.44	5.51	87.22	1.83	98.20	8.17
	EP	ND	96.89	2.60	115.97	1.05	96.54	5.61
	PP	0.06	81.42	5.01	99.33	0.57	93.97	3.30

**Table 6:** Comparison of the developed method with other reported methods for PBs.

Analytical Method	Sample	Analyte	Linearity (µg/L)	LOD (µg/L)	Recovery (%)	Ref.
MIP-OT/HPLC	PCPs ^a^	Six PBs	0.5–600	0.2–0.3	91–103	[29]
U-PLS/RBL/GCE	Sweetener	Four PBs	0.78–4.48 μmol/L	0.29–0.39 μmol/L	82–121	[41]
PDA@MIL101@Fe_3_O_4_@Car d-MSPE/HPLC	Skin cleanser and mouthwash	Four PBs	0.05–1.0	0.05–1.0	80–96	[42]
MIP-SPME/HPLC	Water	MP, EP, PP	2.0–50.0	0.26–0.36	92–99	[43]
SWNHs/HPLC	Urine	Four PBs	5–1000	1–7	105–116	[44]
MEPS/HPLC-MS/MS	Water	Five PBs	0.2–20	0.06–0.09	82–119	[45]
SMIP fiber array/HPLC	Water	MP, EP, PP	0.01–200	0.003–0.02	80–117	This work

^a^ PCPs: Personal care and cosmetic products.

## Data Availability

Not applicable.

## Supplementary Materials

The following supporting information can be downloaded at: https://www.mdpi.com/article/10.3390/ijms231810753/s1.
