# Supplementary material for: β-Cyclodextrin-Based Supramolecular Imprinted Fiber Array for Highly Selective Detection of Parabens
Source: Int J Mol Sci. 2022 Sep 15;23(18):10753. doi: 10.3390/ijms231810753 (PMC9500753; doi:10.3390/ijms231810753)
Supplement: Supplementary file 1 [file ijms-23-10753-s001.zip › ijms-1894712-supplementary.pdf]

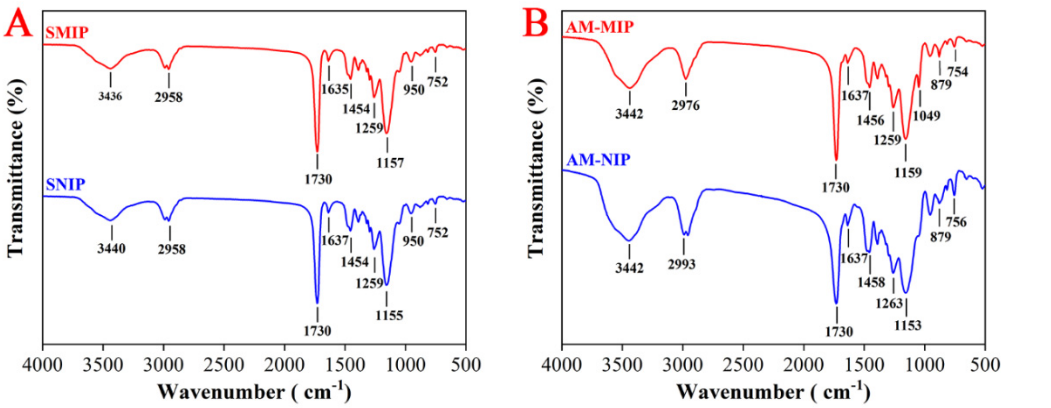


**Figure S1.** FT-IR of imprinted polymers fiber coatings. A). SMIP and SNIP; B). AM-MIP and AM-NIP.

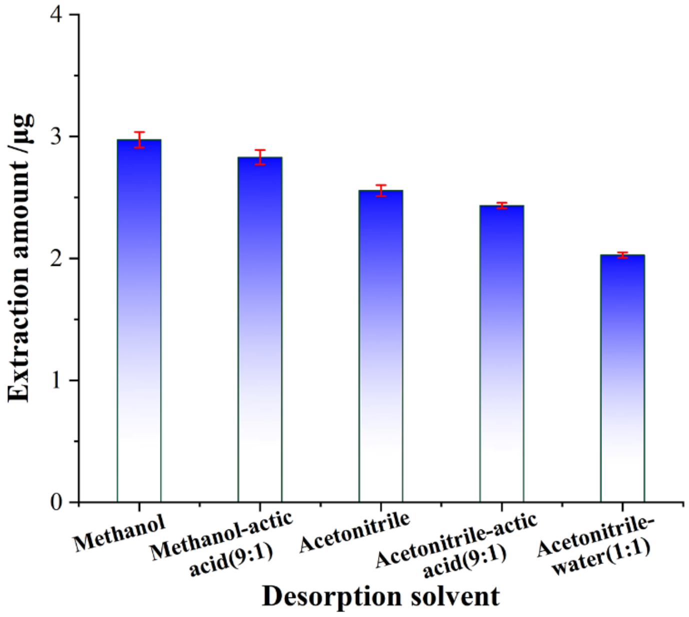


**Figure S2.** Desorption solvent optimization.

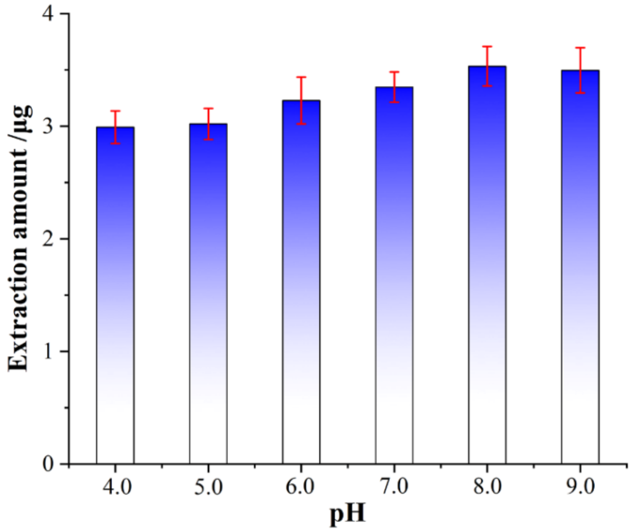


**Figure S3.** pH optimization of extraction solution.

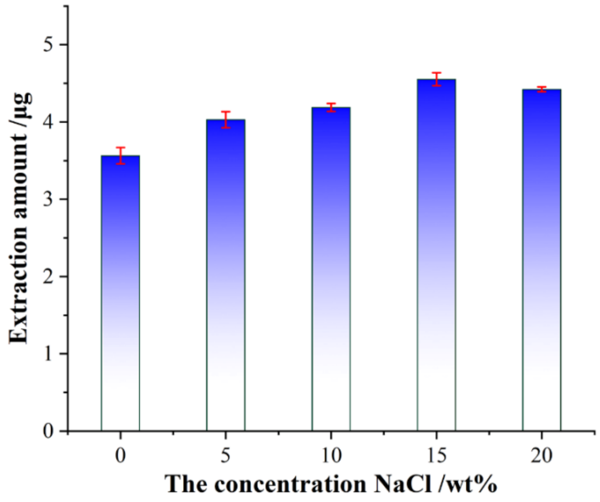


**Figure S4.** Optimization of the ionic strength of the extraction solution.

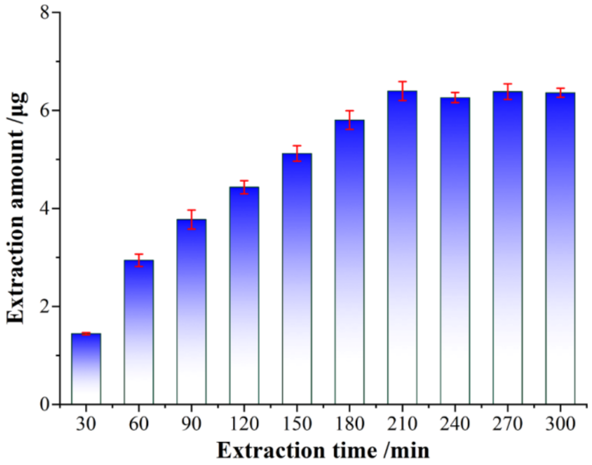


**Figure S5.** Optimization of extraction time.

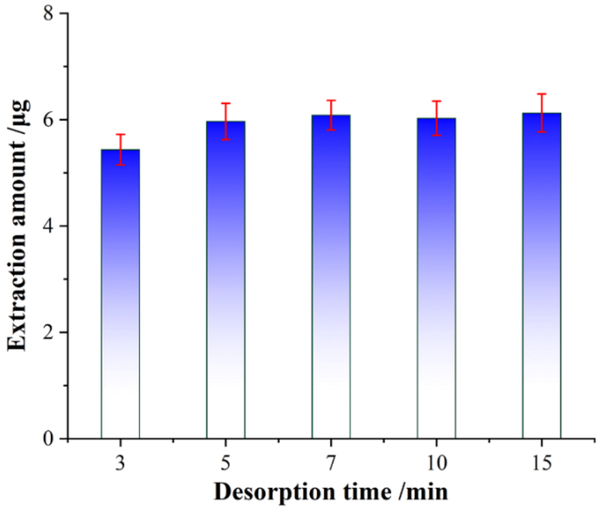


**Figure S6.** Optimization of desorption time.

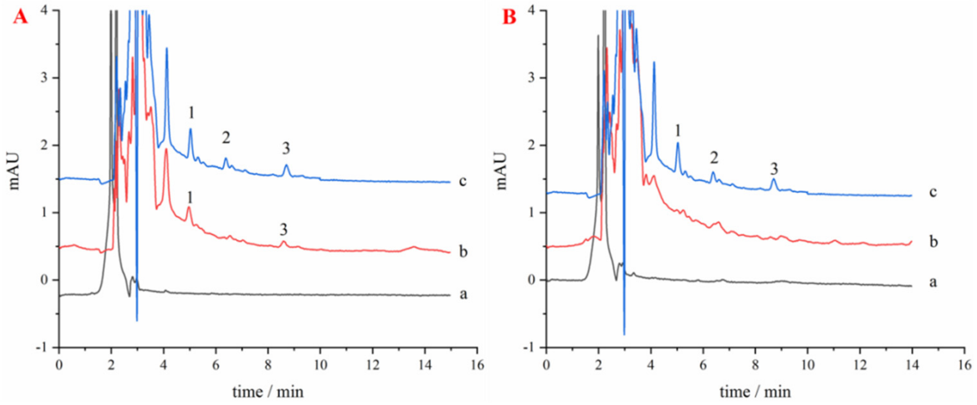


**Figure S7.** Environmental water sample chromatogram. (A) Sampling point 1 environmental water sample; (B) Sampling point 2 environmental water sample; (a) Environmental water sample; (b) Environmental water sample after SMIP fiber array processing; (c) Added 0.1 μg /L environmental water sample after SMIP fiber array processing. 1: MP. 2: EP. 3: PP.

**Table S1.** Brunauer-Emmett-Teller (BET) measures surface area and pore parameters.

| Fibers | Surface area ($m^2/g$) | Average pore Diameter (nm) | Total pore volume ($cm^3/g$) |
|---|---|---|---|
| SMIP | 13.081 | 7.16 | 0.023 |
| NIP | 5.743 | 1.10 | 0.016 |
| AM-MIP | 437.196 | 8.12 | 0.89 |

**Table S2.** Analysis of desorption rate and extraction rate (100 μg/L).

| Analytes | Desorption rate /% | | | Extraction rate /% | | |
|---|---|---|---|---|---|---|
| | SMIP fiber array | SNIP fiber array | AM-MIP fiber array | SMIP fiber array | SNIP fiber array | AM-MIP fiber array |
| MP | 81.54 | 80.96 | 77.81 | 67.55 | 54.19 | 60.01 |
| EP | 77.73 | 77.17 | 86.97 | 75.81 | 73.08 | 64.51 |
| PP | 81.64 | 75.10 | 70.51 | 99.41 | 86.55 | 86.16 |
| Phenol | 89.22 | 87.26 | 86.06 | 8.38 | 20.98 | 11.84 |
| Aniline | 87.37 | 88.17 | 87.94 | 2.71 | 1.70 | 1.53 |

**Table S3.** Evaluation for extraction performance of imprinting materials.

| Fibers | Monomers | Response signal (mAU) | RSD (%) | IF* |
|---|---|---|---|---|
| 1 MIP | mono-(6-mercapto-6-deoxy)-β-CD | 12.0157 | 5.4 | 1.29 |
| 1 NIP | | 9.4570 | 2.7 | |
| 2 MIP | mono-(6-N-propargyl-6-deoxy)-β-CD | 14.5178 | 3.7 | 1.26 |
| 2 NIP | | 11.4840 | 4.3 | |
| 3 MIP | mono-(6-O-p-vinylbenzene sul-fonyl-6-deoxy)-β-CD | 10.2432 | 6.2 | 1.15 |
| 3 NIP | | 8.8834 | 1.5 | |
| 4 MIP | mono-(6-N-diallylamine-6-deoxy)-β-CD | 12.3072 | 2.8 | 1.05 |
| 4 MIP | | 11.7066 | 5.9 | |
| 5 MIP | mono-(6-N-allylamino-6-deoxy)-β-CD | 14.3600 | 4.2 | 1.22 |
| 5 NIP | | 11.7879 | 2.3 | |
| 6 MIP | MAA | 8.7574 | 1.8 | 1.09 |
| 6 NIP | | 7.9770 | 6.6 | |
| 7 MIP | AM | 11.1603 | 1.3 | 1.14 |
| 7 NIP | | 9.7804 | 1.4 | |
| 8 MIP | 4-VP | 8.1142 | 8.8 | 1.08 |
| 8 NIP | | 7.4605 | 7.0 | |

IF* was calculated here by the ratio of response signal of MIP to the response signal of NIP because during the material preparation and extraction optimization process, the quantitative analysis standard curve had not been established.
